# Comparison of operatively and nonoperatively treated isolated Weber B ankle fractures: a systematic review and meta-analysis

**DOI:** 10.1186/s13018-024-04835-4

**Published:** 2024-06-10

**Authors:** Jinhui Tian, Jie Miao, Zhongchao Jiang, Zhiyuan Li

**Affiliations:** Department of Orthopedic Surgery, Handan Central Hospital, 15 South Zhonghua Street, Handan, Hebei, 056008 China

**Keywords:** Weber B, Ankle, Operative, Conservative, Meta- analysis

## Abstract

**Background:**

Despite fractures of Isolated Weber B being prevalent, there is a lack of clarity regarding the relative effectiveness of surgical versus conservative treatment. This systematic review and meta-analysis aimed to investigate the clinical effects and complications of surgical versus conservative treatment of the Isolated Weber B ankle fractures.

**Methods:**

This study involved thorough searches across multiple electronic databases, including PubMed, Cochrane, Embase, and Web of Science, to identify all relevant publications on Isolated Weber B ankle fractures repaired through surgical versus conservative treatment. Through a comprehensive meta-analysis, several outcomes were evaluated, including post-operative function, complications and reoperation rate.

**Result:**

Six articles involving 818 patients who met the inclusion criteria. Among these participants, 350 were male and 636 were female. 651 patients received conservative treatment, while 396 underwent surgical intervention. The findings indicate no significant differences in OMAS, FAOQ, PCS, MCS scores, and return to work between surgical and non-surgical treatments for isolated Weber B ankle fractures. However, compared with surgical treatment, non-surgical treatment has a higher AOFAS score(MD = -5.31, 95% CI = [-9.06, -1.55], *P* = 0.20, I^2^ = 39%), lower VAS score(MD = 0.72, 95% CI = [0.33, 1.10], *P* = 0.69, I^2^ = 0%), lower complication rate (RR = 3.06, 95% CI = [1.58, 6.01], *P* = 0.05, I^2^ = 54%), and lower reoperation rate(RR = 8.40, 95% CI = [1.57, 45.06], *P* = 0.05, I^2^ = 67%).

**Conclusion:**

The meta-analysis does not allow for a definitive recommendation of conservative treatment over surgical intervention for isolated Weber B ankle fractures. While non-surgical approaches may provide comparable functional outcomes and fewer short-term complications, the presence of high bias and limitations in the existing studies calls for caution.

## Introduction

Ankle fractures are a common injury, with a prevalence of one fracture per 800 people [1–3]. Of all the fractures of the lower extremity fractures seen in emergency departments, they comprise 19.2% [4, 5]. The most common ankle fracture is the isolated trans-syndesmotic fibula fracture [1]. It is also known as a Weber B or type 44B under the AO (Association for the Study of Internal Fixation) and OTA (Orthopaedic Trauma Association) classifications [6–9].

For AO 44-B ankle fractures, treatment strategies encompass either surgical stabilization through internal fixation with plates and screws or conservative management employing casts or walking boots [2]. Supporters of surgical treatment for the 44-B ankle fracture stress the necessity of achieving anatomical precision with internal fixation to minimize the chances of displacement and ensure stability [2, 10, 11]. This approach is particularly favored in patient populations such as athletes or those with high physical demands, as surgical intervention is believed to provide a quicker and more reliable return to full function. The hypothesis is that surgical fixation provides better anatomical restoration, reduces the risk of displacement, and allows for earlier mobilization compared to conservative methods. Moreover, some studies suggest that surgical intervention may lead to better long-term functional outcomes and lower rates of malunion and nonunion, which are critical considerations for active individuals.

Conversely, it is believed that surgical interventions are associated with higher costs and an increased likelihood of adverse outcomes [10, 12–14]. . These include standard risks related to anesthesia and surgical procedures, such as mortality, venous thromboembolism, infections, failures in fixation, and the potential necessity for subsequent corrective surgeries [13]. Non-operative approaches are argued to yield favorable long-term results without exposing patients to the inherent risks of an operative procedure. For patients with lower physical demands or those with comorbidities that increase surgical risks, conservative treatment may be the preferred option. The hypothesis supporting conservative management argues that, in many cases, the outcomes in terms of pain relief and functional recovery are comparable to those achieved with surgery. Although randomized controlled studies have demonstrated that surgical approaches are not superior to conservative management for treating Weber B ankle fractures, a comprehensive meta-analysis on this topic is absent. This meta-analysis aims to incorporate research on conservative and surgical treatments for Weber B fractures and investigate the clinical effects and complications of two treatment methods.

## Materials and methods

### Search strategy

This study adhered to the PRISMA (Preferred Reporting Items for Systematic Reviews and Meta-Analyses) guidelines in its reporting of systematic reviews and meta-analyses [15]. The review protocol was registered in the PROSPERO database CRD42024522834 [16]. The last search date was March 5, 2024, and the PubMed, the Cochrane Library, Embase and Web of Science databases were searched. The search procedure is based on the following keywords: (“Isolated lateral malleolus fracture*” OR “Isolated fibula*” OR “Weber B” OR “type B”) AND (“Surg*” OR “operati*” OR “ORIF” OR “open reduction and internal fixation”) AND (“non-surg*” OR “no surg*” OR “nonsurg*” OR “non-operati*” OR “no operati*” OR “nonoperati*” OR “conservative” OR “cast” OR “brace”). The title and abstract of the articles were initially examined to decide if they should be included. Next, the full articles that met the initial criteria were read. Moreover, the references for those articles were checked to ensure the search was complete.

### Inclusion and exclusion criteria

People between 18 years and above who had isolated type B fibula fractures without significant talar shift identified radiographically were eligible for inclusion. Exclusion criteria were patients with medial malleolus or posterior malleolus fracture, deltoid ligament injury, syndesmotic injury (assessed using weight-bearing X-rays), or fixation, as well as those with open fractures, polytrauma, subsequent ankle trauma, medical comorbidities preventing weight bearing, severe pre-existing ankle arthritis, or skeletal immaturity. Surgical treatment was considered the experimental group, while conservative treatment was the control intervention. Outcomes measured included functional scores (AOFAS, OMAS, FAOQ, PCS, MCS, VAS), return to work, complication, and reoperation rate. Randomized controlled trials, retrospective cohort studies, and prospective cohort studies were included to provide a comprehensive analysis of the available evidence. Case reports, reviews, and studies with low homogeneity were excluded. The study imposed a language restriction, including only studies published in English.

### Quality assessment

Two researchers conducted a quality assessment of the RCTs using the Cochrane Reviewer’s Handbook [17], examining seven specific domains of bias: the method of random sequence generation, the concealment of allocation process, blinding practices for both participants and staff, blinding during outcome evaluation, completeness of reported outcomes, the presence of selective outcome reporting, and potential other biases. Each domain was rated on a scale of low, unclear, or high risk of bias. Furthermore, two independent reviewers (J.H.T. and J.M.) evaluated the methodological quality of the studies included in this research, using the Methodological Index for Non-Randomized Studies (MINORS) [18]. This validated tool is specifically designed to assess the methodological quality and clear reporting of observational surgical studies [18]. Any disagreements were resolved by consulting a third reviewer (Z.Y.L.)

### Data extraction

Two independent reviewers systematically extracted critical data from selected studies, including the name of the first author, publication year, number of participants, demographic details (age and gender), treatment type, and follow-up duration. We evaluated outcomes based on AOFAS, OMAS, FAOQ, PCS, MCS, VAS scores, complication and reoperation rate. To address the absence of standard deviations in the data, the study authors were emailed for additional information. Following the guidance of Wan et al. [19], medians and ranges were converted to approximate means and standard deviations (SDs).Any disagreements encountered during the data extraction phase were resolved by consulting a third investigator (Z.Y.L.).

### Statistical analysis

Review Manager (RevMan version 5.4.1, Nordic Cochrane Center, Copenhagen, Denmark) was utilized to analyze the data. Continuous variables were assessed using the mean difference (MD) accompanied by 95% confidence intervals (CIs), and risk ratios (RR) with 95% CIs were employed for the analysis of dichotomous variables. The presence of heterogeneity was evaluated through I2 tests, where I^2^ values exceeding 50% and P-values less than 0.05 were indicative of substantial heterogeneity, applying the adoption of random-effects models. For lower heterogeneity, fixed-effect models were applied. Sensitivity analysis was performed by systematically omitting one study at a time to assess the impact of each individual study on the overall pooled results and to ensure the robustness and stability of the findings. This method helps identify if any single study disproportionately influences the meta-analysis outcomes.

## Result

### Study selection

**Fig. 1:**
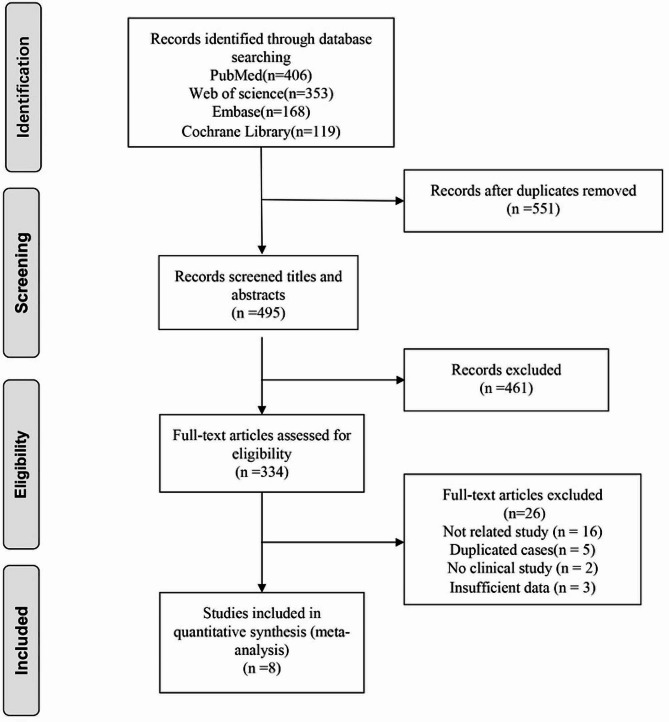
Flow diagram of screening the included studies in the meta-analysis

The PRISMA flow diagram (Fig. 1) illustrates the literature search and selection process. A total of 1046 relevant articles were identified by searching electronic databases. 6 articles were selected for this meta-analysis, all of which compared surgical and nonoperative treatment of isolated lateral malleolus fractures.

### Study characteristics

The meta-analysis included 8 studies comparing surgical and nonoperative treatment of isolated lateral malleolus fractures. These studies included 2 randomized controlled trials, 2 prospective, and 4 retrospective cohort studies, with a total of 818 patients (257 men and 500 women), of which 297 was treated surgically and 521 was treated conservatively (Table 1).

**Table 1:** General characteristics of the included studies

Author and year	study design	Treatment	Number	Male/female	Age (years)	BMI (Kg/m^2^)	Follow-up(months)
Laurence 2024 [20]	RCS	Non-operative	29	11/18	58.2 ± 13.8	28.7 ± 5.3	80.4 ± 9.6
		Operative	20	6/14	55.0 ± 14.4	28.1 ± 5.9	82.8 ± 39.6
O’Keefe 2022 [21]	RCT	Non-operative	37	16/21	42.8 ± 13.8	28.6 ± 6.4	≥ 60
		Operative	40	20/20	37.1 ± 12.7	28.0 ± 5.4	≥ 60
van Leeuwen② 2019 [22]	RCS	Non-operative	17	13/4	60.6 (35–95)	NA	70.5 (31–106)
		Operative	39	18/21	50.2 (25–80)	NA	55.4 (22–102)
van Leeuwen③ 2019 [22]	RCS	Non-operative	25	14/11	64.4 (24–95)	NA	59.7 (20–106)
		Operative	68	33/35	56.2 (25–86)	NA	29.7 (0-106)
Mittal① 2017 [23]	RCS	Non-operative	38	21/17	43 ± 14	NA	24
		Operative	8	2/6	39 ± 17	NA	24
Mittal② 2017 [24]	RCT	Non-operative	80	41/39	39.8 ± 13.7	28.4 ± 6.6	12
		Operative	80	42/38	38.1 ± 13.0	27.7 ± 5.2	12
Mittal③ 2017 [24]	PCS	Non-operative	257	15/242	39.4 ± 13.7	27.6 ± 5.5	12
		Operative	19	5/14	31.1 ± 11.5	26.2 ± 2.9	12
Dietrich 2002 [25]	PCS	Non-operative	38	NA	NA	NA	1.73 (0.5-6)
		Operative	23	NA	NA	NA	6.2 (0.5–16)

Note: RCS: Retrospective cohort study; RCT: randomized controlled trial; PCS: prospective cohort study;

### Sensitivity analysis and risk of bias assessment

**Fig. 2:**
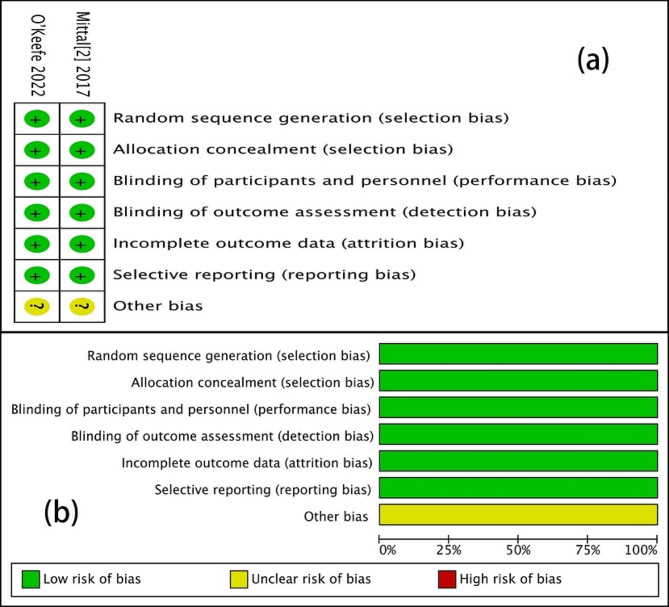
Review authors’ judgments about each risk of bias item for each included study. (**a**) Risk of bias summary; (**b**) risk of bias graph presented as percentages

The robustness of the results was assessed by performing sensitivity analyses. In most results, the heterogeneity results did not change significantly after omitting each study in turn, suggesting that our results are statistically robust. The risk of bias items for each included study is shown in Fig. 2. Furthermore, Table 2 shows the study quality distribution of non-randomized controlled studies. The mean (± SD) MINORS score was 19.2 ± 0.75 (range: 18–20).

**Table 2:** Risk of bias assessment for non-randomized studies using the MINORS tool. Item scores are 0 (not reported), 1 (reported but inadequate) or 2 (reported and adequate). Quality assessment for the six cohort studies according to Newcastle-Ottawa scale (NOS)

MINORS Criteria:	Laurence		van Leeuwen②	van Leeuwen③	Mittal①	Mittal③	Dietrich
Clearly Stated Aim	2		2	2	2	2	2
Consecutive Patients	2		2	2	2	2	2
Prospective Data Collection	1		2	2	1	2	1
Appropriate Endpoints	2		2	2	2	2	2
Unbiased Endpoint Assessment	2		2	2	2	2	2
Appropriate Follow-up	2		2	2	2	2	1
Loss to Follow-Up < 5%	0		2	2	2	2	2
Prospective Calculation of Study Size	0		0	0	0	0	0
Adequate Control Group	2		1	1	0	1	2
Contemporary Groups	2		2	2	1	1	1
Baseline Equivalence of Groups	2		1	1	2	1	2
Adequate Statistical Analyses	2		2	2	2	2	2
Total Points (x/24)	19		20	20	18	19	19

Note: S selection, C comparability, E exposure. S1 representativeness of the exposed cohort, S2 selection of the nonexposed cohort, S3 ascertainment of exposure, S4 demonstration that outcome of interest was not present at start of study. C1 comparability of controls for the most important factor, C2 comparability of controls for a second important factor. E1 assessment of outcome, E2 was follow-up long enough for outcomes to occur, E3 adequacy of follow-up of cohorts

### Functional outcome

#### AOFAS score

**Fig. 3:**
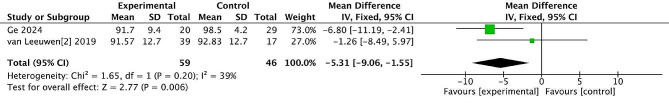
Post-treatment AOFAS score

Two studies reported post-treatment AOFAS scores, with 59 patients treated surgically and 46 patients treated conservatively. The surgical and non-surgical groups had post-treatment AOFAS scores of 91.6 and 95.6, respectively. There are significant differences between the two treatments (MD = -5.31, 95% CI = [-9.06, -1.55], *P* = 0.006), with low heterogeneity (*P* = 0.20, I^2^ = 39%). (Fig. 3)

#### OMAS score

**Fig. 4:**
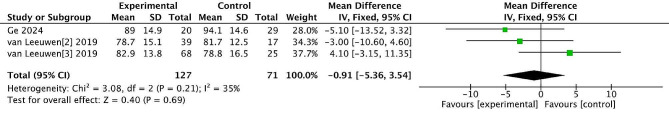
Post-treatment OMAS score

Two studies reported post-treatment OMAS scores, with 127 patients treated surgically and 71 patients treated conservatively. The surgical and non-surgical groups had post-treatment OMAS scores of 83.5 and 84.9, respectively. There are no significant differences between the two treatments (MD = -0.91, 95% CI = [-5.36, 3.54], *P* = 0.69), with low heterogeneity (*P* = 0.21, I^2^ = 35%). (Fig. 4)

#### FAOQ score

**Fig. 5:**
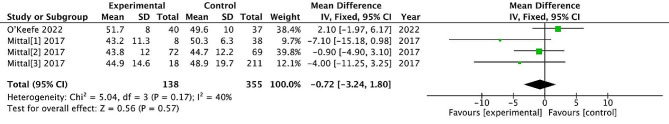
Post-treatment FAOQ score

Four studies reported post-treatment FAOQ scores, with 138 patients treated surgically and 355 patients treated conservatively. The surgical and non-surgical groups had post-treatment FAOQ scores of 45.9 and 48.4, respectively. There are no significant differences between the two treatments (MD = -0.72, 95% CI = [-3.24, 1.80], *P* = 0.57), with low heterogeneity (*P* = 0.17, I^2^ = 40%). (Fig. 5)

#### PCS score

**Fig. 6:**
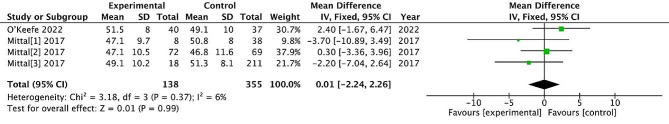
Post-treatment PCS score

Three studies reported post-treatment PCS scores, with 138 patients treated surgically and 355 patients treated conservatively. The surgical and non-surgical groups had post-treatment PCS scores of 45.9 and 48.4, respectively. There are no significant differences between the two treatments (MD = 0.01, 95% CI = [-2.24, 2.26], *P* = 0.99), with low heterogeneity (*P* = 0.37, I^2^ = 6%). (Fig. 6)

#### MCS score

**Fig. 7:**
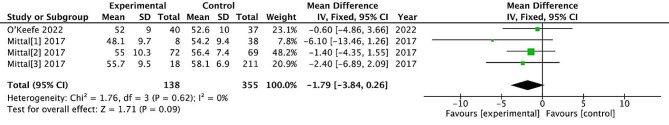
Post-treatment MCS score

Three studies reported post-treatment MCS scores, with 138 patients treated surgically and 355 patients treated conservatively. The surgical and non-surgical groups had post-treatment MCS scores of 52.7 and 55.3, respectively. There are no significant differences between the two treatments (MD = -1.79, 95% CI = [-3.84, 0.26], *P* = 0.09), with low heterogeneity (*P* = 0.62, I^2^ = 0%). (Fig. 7)

#### VAS score

**Fig. 8:**
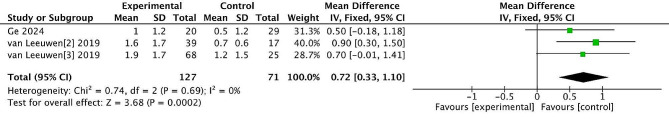
Post-treatment VAS score

Three studies reported post-treatment VAS scores, with 127 patients treated surgically and 71 patients treated conservatively. The surgical and non-surgical groups had post-treatment VAS scores of 1.5 and 0.8, respectively. There are significant differences between the two treatments (MD = 0.72, 95% CI = [0.33, 1.10], *P* = 0.0002), with low heterogeneity (*P* = 0.69, I^2^ = 0%). (Fig. 8)

### Return to work

**Fig. 9:**
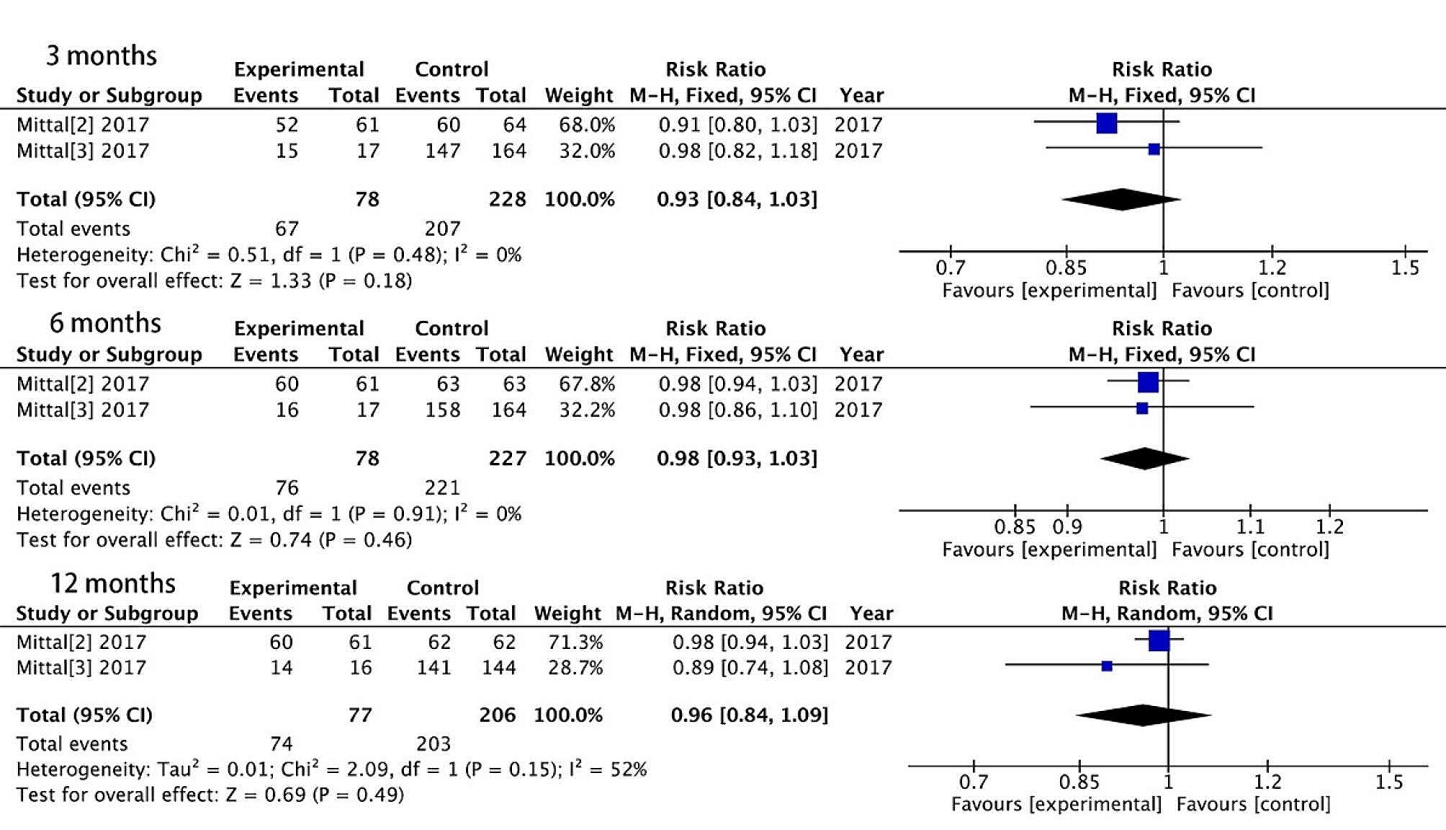
Return to work

Two studies reported a return to work, with 78 patients treated surgically and 228 patients treated conservatively. The rates of return to work 3 months after treatment were 85.9% and 90.8% in the surgical and non-surgical groups, respectively. There are no significant differences between the two treatments (RR = 0.93, 95% CI = [0.84, 1.03], *P* = 0.18), with no heterogeneity (*P* = 0.48, I^2^ = 0%). The rates of return to work 6 months after treatment were 97.4% and 97.4% in the surgical and non-surgical groups, respectively. There are no significant differences between the two treatments (RR = 0.98, 95% CI = [0.93, 1.03], *P* = 0.46), with no heterogeneity (*P* = 0.91, I^2^ = 0%). The rates of return to work 12 months after treatment were 96.1% and 98.5% in the surgical and non-surgical groups, respectively. There are no significant differences between the two treatments (RR = 0.96, 95% CI = [0.84, 1.09], *P* = 0.49), with high heterogeneity (*P* = 0.15, I^2^ = 52%). A random effects model was used to deal with heterogeneity. (Fig. 9)

### Complication

#### Total complication

**Fig. 10:**
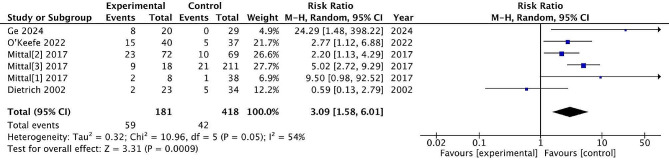
Total complication

Six studies reported post-treatment total complication, with 181 patients treated surgically and 418 patients treated conservatively. The incidence of total complication rate was 32.6% and 10.0% in the surgical and non-surgical groups, respectively. There are significant differences between the two treatments (RR = 3.06, 95% CI = [1.58, 6.01], *P* = 0.0009), with high heterogeneity (*P* = 0.05, I^2^ = 54%). (Fig. 10) A random effects model was used to deal with heterogeneity.

#### Nerve injury

**Fig. 11:**
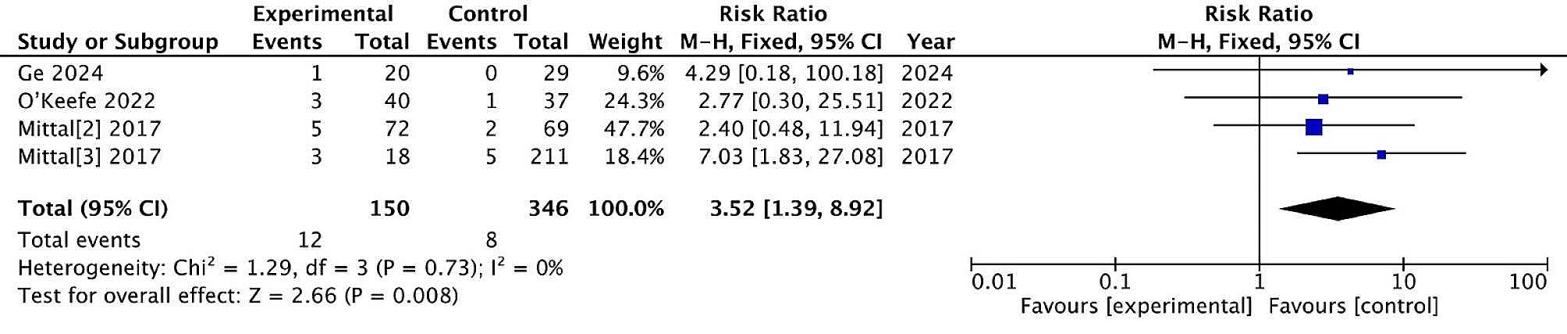
Nerve injury

Four studies reported post-treatment nerve injury, with 150 patients treated surgically and 346 patients treated conservatively. The incidence of nerve injury was 8.0% and 2.3% in the surgical and non-surgical groups, respectively. There are significant differences between the two treatments (RR = 3.52, 95% CI = [1.39, 8.92], *P* = 0.0008), with no heterogeneity (*P* = 0.73, I^2^ = 0%). (Fig. 11)

#### Wound complication

**Fig. 12:**
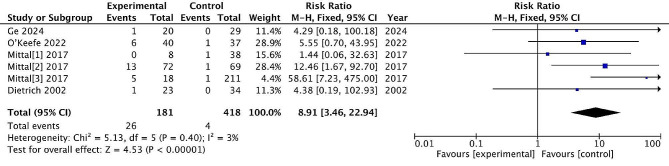
Wound complication

Six studies reported post-treatment wound complication, with 181 patients treated surgically and 418 patients treated conservatively. The incidence of wound complication rate was 14.3% and 0.9% in the surgical and non-surgical groups, respectively. There are significant differences between the two treatments (RR = 8.91, 95% CI = [3.46, 22.94], *P* < 0.00001), with low heterogeneity (*P* = 0.40, I^2^ = 3%). (Fig. 12)

#### Major infection

**Fig. 13:**
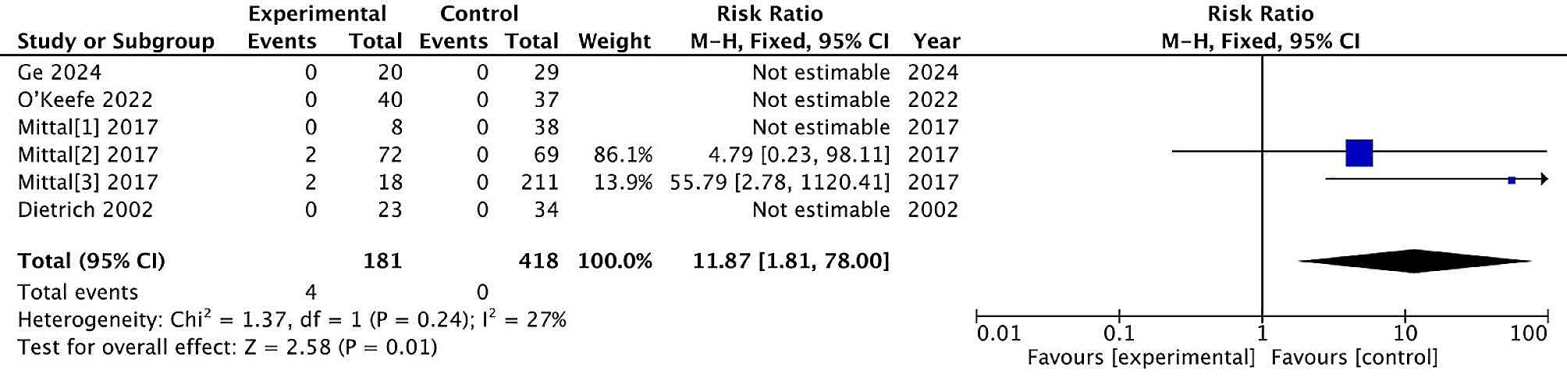
Major infection

It is a major infection requiring surgical intervention, hospitalization, or intravenous antibiotics. Examples include deep surgical site infections and osteomyelitis. Six studies reported post-treatment major infection, with 181 patients treated surgically and 418 patients treated conservatively. The incidence of major infection rate was 2.2% and 0% in the surgical and non-surgical groups, respectively. There are significant differences between the two treatments (RR = 11.87, 95% CI = [1.81, 78.00], *P* = 0.01), with low heterogeneity (*P* = 0.24, I^2^ = 27%). (Fig. 13)

#### Minor infection

**Fig. 14:**
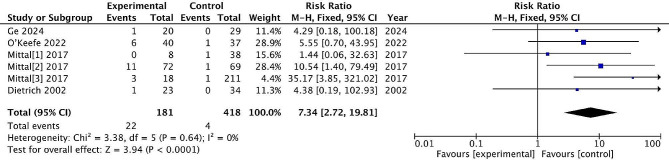
Minor infection

It is a minor infection that can be managed with oral antibiotics and does not require surgical intervention or hospitalization. Examples include superficial wound infections. Six studies reported post-treatment minor infection, with 181 patients treated surgically and 418 patients treated conservatively. The incidence of minor infection rate was 12.2% and 0.96% in the surgical and non-surgical groups, respectively. There are significant differences between the two treatments (RR = 7.34, 95% CI = [2.72, 9.81], *P* < 0.0001), with no heterogeneity (*P* = 0.64, I^2^ = 0%). (Fig. 14)

#### DVT

**Fig. 15:**
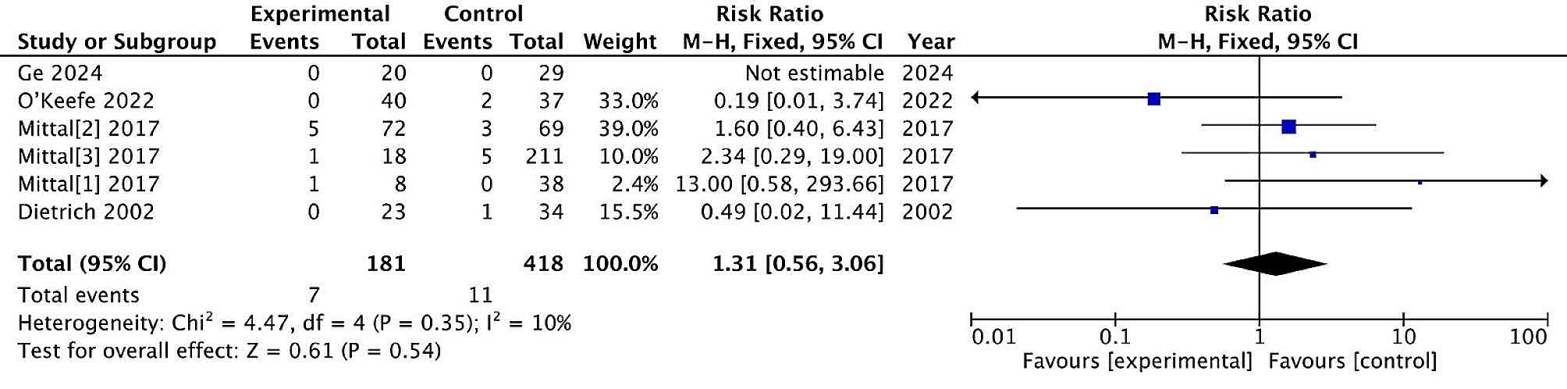
DVT

Six studies reported post-treatment DVT, with 181 patients treated surgically and 418 patients treated conservatively. The incidence of DVT rate was 3.9% and 2.6% in the surgical and non-surgical groups, respectively. There are no significant differences between the two treatments (RR = 1.31, 95% CI = [0.56, 3.06], *P* = 0.54), with low heterogeneity (*P* = 0.35, I^2^ = 10%). (Fig. 15)

#### Reoperation

**Fig. 16:**
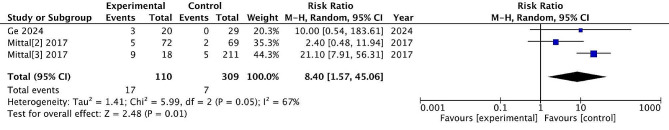
Reoperation

The primary reasons for reoperation in the surgical group were related to implant symptoms, such as irritation or pain at the implant site. Three studies reported post-treatment reoperation rate, with 110 patients treated surgically and 309 patients treated conservatively. The incidence of reoperation rate was 15.5% and 2.3% in the surgical and non-surgical groups, respectively. There are significant differences between the two treatments (RR = 8.40, 95% CI = [1.57, 45.06], *P* = 0.01), with high heterogeneity (*P* = 0.05, I^2^ = 67%). (Fig. 16) A random effects model was used to deal with heterogeneity.

## Discussion

The outcomes of the study suggest that non-surgical and surgical treatments yield comparable outcomes in functional scores such as OMAS, FAOQ, PCS, and MCS. However, non-surgical treatment is associated with higher AOFAS and lower VAS scores. Furthermore, while the rate of return to work and occurrence of deep vein thrombosis (DVT) is similar between the two treatment modalities, surgical management presents an increased incidence of nerve injury and wound complications.

The results show that nonsurgical treatment can achieve good clinical results and have a lower complication rate compared with surgical treatment. Stockwell et al. conducted a six-month prospective study on 62 patients suffering from isolated OTA/AO 44B fractures who underwent non-surgical treatment. The findings revealed that patients achieved an excellent outcome, as evidenced by their AOFAS scores [26]. The findings also showed that AOFAS scores are significantly higher for patients receiving non-surgical treatment than those undergoing surgical intervention. In a randomized controlled trial, Kortekangas et al. applied conservative management to patients with stable Weber B fractures, achieving an Olerud-Molander Ankle Score (OMAS) of 91.7 at a 52-week follow-up period [27].A retrospective study conducted by Van Laarhoven et al. [28], which involved 579 patients over a median observation period of five years, advocated for a wide-ranging approach to conservative treatments, including functional methods like tape bandaging. The findings supported a limited use of implants in osteosynthesis as being reasonable. Stassen et al. [29] applied a walking boot as a method of conservative treatment for 50 patients diagnosed with stable Weber B fractures, achieving satisfactory outcomes. Evidence from both randomized and non-randomized controlled trials suggests that conservative management of stable ankle fractures yields favorable outcomes in the short and long term. In contrast, surgical treatment of distinctly unstable ankle fractures leads to enhanced outcomes [30–32]. Abdelaal et al. believed that in the absence of any clinical or radiographic signs of instability, isolated Weber B trans-syndesmotic fractures can be safely managed through the application of functional bracing and the early initiation of weight bearing [33]. Rooney et al. propose that isolated Weber B fibular fractures, evidenced by less than 7 mm of MCS widening on weight-bearing X-rays and a discrepancy of less than 2 mm compared to the contralateral ankle, are suitable for conservative treatment [34]. Some studies suggest no significant difference in short-term and long-term functional outcomes for isolated lateral malleolar fractures between surgical and non-surgical treatments [24, 35–37]. Sanders applied stress position X-ray analysis to Weber type B ankle injuries and found results similar to those of the present study. Specifically, they reported no significant functional differences between surgical and nonsurgical treatment modalities [35].

The study results show that surgical treatment did not provide enhanced functional benefits over conservative treatment. Additionally, the complication rates for surgical interventions were significantly elevated compared to non-surgical treatments. The systematic review by Donken et al. [2] indicated insufficient evidence to warrant surgery for type B ankle fractures. This was attributed to the fact that the RCTs highlighted in the review involved patients with different ankle fracture patterns or with significant talar shifts, which could influence the necessity for surgical treatment [9, 31, 32, 38–40]. Magan et al. stated in a systematic review that syndesmotic screws were used in 40% of Weber B type ankle fractures in order to maintain the stability of the ankle joint [1]. However, several other studies have indicated that the efficacy of surgical interventions may not surpass that of conservative functional therapies, a conclusion that aligns with the outcomes observed in this research. In a year-long follow-up study by Sanders et al. [35], involving patients with slight talus displacement, findings indicated that the functional outcomes of surgical treatment were not superior to those obtained through conservative management.

The study indicates that the rate of return to work at post-treatment 3, 6, and 12 months was similar between the conservative and surgical treatment groups. This suggests that both treatment modalities allow for a satisfactory functional recovery regarding work-related activities. However, Ponzer et al. [41] observed that among patients treated surgically for a single Weber-B fracture, only 36% reported a complete recovery, with 44% experiencing work-related issues and 61% facing difficulties with sports activities. Additionally, a recent meta-analysis showed that conservative treatment of ankle fractures resulted in a faster return to sports activities [42]. These results highlight the variability in recovery outcomes and underscore the importance of discussing recovery expectations with patients, particularly those engaged in high-demand physical activities. It is essential to tailor discussions based on individual patient profiles and treatment choices, as these factors can significantly influence their ability to return to play and overall quality of life.

The study indicates that the incidence rate of complications associated with conservative management is 10.0%, in contrast to a 32.6% complication rate observed in surgical interventions. Gougoulias et al. found that among 213 stable fractures treated nonoperatively, 2.8% of the ankles developed radiographic osteoarthritis (mean follow-up of 18 years) and 84% of the ankles were asymptomatic [43]. Beauchamp et al. argued that internal fixation for ankle fractures in women over the age of 50 is correlated with a high incidence of complications, offering limited advantages [44]. Several studies have indicated that the surgical stabilization of lateral malleolar fractures is associated with a high risk of adverse events, leading to the necessity for implant removal surgeries due to discomfort [22, 24]. In a retrospective analysis conducted by Frederiksen et al. [45], surgical intervention for stable isolated lateral malleolus fractures was found to be associated with a significant risk of severe adverse events. Additionally, it was observed that 33.3% of patients (*n* = 36) required subsequent procedures for the removal of implants. In a prospective study by Wenger et al. [46], the risk of reoperation was 24% when AO/OTA 44-B fractures were treated with lateral plating and 13% when treated with posterolateral plating. In a randomized study by Sanders et al. [35], 81 patients were allocated to receive either surgical treatment or non-operative management. Of the 41 patients in the surgical group, there was a 2.4% incidence (one patient) of deep wound infections requiring surgical revision, 9.8% (four patients) developed superficial infections, and another 9.8% needed implant removal. The study results showed that the reoperation rate of non-surgical treatment was 1.6%, significantly lower than the 23.9% of the surgical group. Richardson et al. [47] conducted a retrospective analysis, finding that 10.7% of patients undergoing open reduction and internal fixation (ORIF) for lateral malleolar fractures experienced surgical site infections within the first year post-operation. The study results show that the incidence rate of wound complications for non-surgical treatment is 10%, while the complication rate for surgical treatment is 0.7%, significantly higher than conservative treatment.

Although the results indicate that conservative management effectively treats isolated Weber B ankle fractures with a lower complication rate, a significant number of patients still opt for surgical intervention. This preference can be attributed to several factors. Javad et al. suggest that surgery outperforms conservative management by preventing early treatment failures, achieving more anatomical reductions, reducing the incidence of malunion and nonunion, and lowering readmission rates [2]. Additionally, Willett et al. reported a high rate of early treatment failure (26%) in the close contact casting group, primarily due to the inability to achieve or maintain reduction [3]. Furthermore, Keene et al. found a higher early failure rate in conservatively treated unstable ankle fractures during a three-year follow-up; however, they also noted that this approach might be acceptable to avoid the initial risks associated with surgical procedures [4].

The main benefits of non-surgical treatment are mainly the avoidance of adverse events and the reduction of related costs [22]. Surgical treatment requires more costs than conservative treatment [48]. In a prospective study, Noback et al. [49] evaluated operative and nonoperative treatment costs in patients with ankle fractures. They found that the direct and indirect costs of operative treatment were higher than those of nonoperative treatment.

The meta-analysis also has some limitations. First, this study only included two RCTs lacking of more high-quality RCT studies. This study suggests that future multicenter randomized controlled studies are needed to draw more definite conclusions. Second, this meta-analysis incorporates studies with differing lengths of follow-up, potentially introducing a source of heterogeneity. Third, the meta-analysis identified the lack of standardization among studies as a significant confounding factor. The definitions of isolated Weber type B ankle fractures in the included studies were different, but they were all considered stable ankle fractures. Fourth, the included studies did not mention the details of fixation type during surgical treatment, which may affect the results. Fifth, the included studies assessed functional outcomes at inconsistent time points, which could also affect the results. Sixth, it is essential to acknowledge the potential bias introduced by combining RCTs and cohort studies in a single analysis. To mitigate this, we applied rigorous quality assessment and sensitivity analyses to evaluate the robustness of the findings. Despite these efforts, the potential for bias remains a limitation of this study.

## Conclusion

The meta-analysis does not allow us to definitively recommend conservative treatment over surgical intervention for isolated Weber B ankle fractures. While non-surgical approaches may provide comparable functional outcomes and fewer short-term complications, the presence of high bias and limitations in the existing studies calls for caution.

## Data Availability

All data generated or analysed during this study are included in this article.
